# The Involvement of TLR2 and TLR4 in Cytokine and Nitric Oxide Production in Visceral Leishmaniasis Patients before and after Treatment with Anti-Leishmanial Drugs

**DOI:** 10.1371/journal.pone.0117977

**Published:** 2015-02-23

**Authors:** Mariana Gatto, Mariana Miziara de Abreu, Karen Ingrid Tasca, Marjorie de Assis Golim, Laura Denise Mendes da Silva, José Cláudio Simão, Carlos Magno Castelo Branco Fortaleza, Ângela Maria Victoriano de Campos Soares, Sueli Aparecida Calvi

**Affiliations:** 1 Tropical Diseases Department, Botucatu School of Medicine—UNESP, Botucatu, São Paulo, Brazil; 2 Flow Cytometry Laboratory, Hemocenter, Botucatu School of Medicine—UNESP, Botucatu, São Paulo, Brazil; 3 Ward of Infectious Diseases, State Hospital of Bauru, Bauru, São Paulo, Brazil; 4 Microbiology and Immunology Department, Biosciences Institute—UNESP, Botucatu, São Paulo, Brazil; University of Leuven, Rega Institute, BELGIUM

## Abstract

Toll-like receptors (TLRs) have significant involvement in *Leishmania* infection, although little is known about the relationship between these receptors, cytokines and nitric oxide (NO) in patients with visceral leishmaniasis (VL) before or after treatment with anti-leishmanial drugs. The goal of this study was to evaluate the expression of TLR2 and TLR4 in CD3^+^ and CD14^+^ cells and the production of TNF-α, IFN-γ, IL-17, IL-10, TGF-β and NO in peripheral blood mononuclear cells (PBMCs) from VL patients pre- and post-treatment with anti-leishmanial drugs. In addition, we investigated whether these receptors were involved in the production of these cytokines and NO. In the active VL patients, increased TLR2 and TLR4 expression in lymphocytes and monocytes, increased production of TNF-α, IL-10 and TGF-β and decreased production of IFN-γ, IL-17 and NO were observed. After treatment, TLR2 and TLR4 were still expressed in lymphocytes and monocytes, the TNF-α and IL-10 levels were lower, the production of IFN-γ, IL-17 and NO was higher, and the TGF-β level remained high. Before treatment, the production of TNF-α and NO was associated with TLR2 and TLR4 expression, while IL-10 production was only associated with TLR2 expression. After treatment, both receptors were associated with the production of TNF-α, IFN-γ, IL-10 and NO, while the production of IL-17 was associated only with TLR4 expression. The results presented in this study suggest that both TLR2 and TLR4 participate in the modulation of cytokine and NO production in VL patients, contributing to the pathogenesis of VL prior to treatment and the protective immune response after treatment.

## Introduction

Visceral leishmaniasis (VL), also known as Kala Azar, is caused by infection with obligate intracellular protozoa belonging to the *Leishmania donovani* complex [1,2]. This disease is fatal if left untreated, and its symptoms include hepatosplenomegaly, fever, anemia, weight loss, hyperglobulinemia and pancytopenia [3]. VL is endemic in 62 countries, and 90% of cases occur in Brazil, Bangladesh, India, Nepal and Sudan [4,5].

Regarding VL treatment, pentavalent antimonials represent the first choice of treatment in Brazil [6,7]. Amphotericin B can be used in cases of toxicity or in patients with poor responses to antimonial therapy, and this drug is also the first choice for treating pregnant patients and terminal cases of VL disease [8,9]. According to the guidelines of The Brazilian Ministry of Health, patients older than 50 years, patients with Chagas disease or patients diagnosed with renal, hepatic or cardiac complications should be treated with colloidal or lipid formulations of amphotericin B [7].

Several lines of evidences indicate an important role for host immune response during infection with *Leishmania* and a large number of studies have aimed to understand the various mechanisms that lead infected individuals to become resistant to or develop VL. However, a complete understanding of these mechanisms still requires further studies. Several studies have shown that the protection of the host in cases of VL depends on the development of a Th1-type response [10,11], whereby interleukin-12 (IL-12) secreted by antigen-presenting cells activates CD4^+^ T cells and Natural Killers (NK) cells to produce interferon-γ (IFN-γ). This cytokine is extremely important for macrophage activation and consequent release of NO, the major metabolite involved in the destruction of parasite. Actived macrophages also secrete tumor necrosis factor-α (TNF-α), which acts in synergy with IFN-γ to activate the microbicidal capacity of these cells [11–14]. It is well known that during active VL, cell-mediated immune responses are supressed and consequently a decrease in IFN-γ [15,16], which is related to the production of regulatory cytokines such as interleukin-10 (IL-10) and tumor growth factor-β (TGF-β) [17–20]. Another cytokine that has been shown to play an important role in leishmaniasis is interleukin-17 (IL-17), produced by Th17 cells, which has pro-inflammatory properties and induces inflammatory mediators [21–24]. Studies on cutaneous leishmaniasis have shown that this cytokine contributes to the pathogenesis of disease [25,26]. In relation to VL, patients from Brazil and Bangladesh have been reported to present higher levels of IL-17 in their serum in comparison with control subjects [27]. *L. donovani* infection leads to a greater Th17 cell differentiation and higher levels of IL-17, which is associated with protection [28]. However, although data show that IL-17 is linked to protection, its role in human VL is not well established and further studies are needed to definitely establish the role of IL-17 in VL.

The main factors that determine the type of immune response that will be triggered is the pattern-recognition receptors (PRRs), which mediate the recognition of microbial structures known as pathogen-associated molecular patterns (PAMPs) and induce inflammatory and adaptive responses [10]. Among the PRRs, the Toll-like receptors (TLRs) are highlighted [10]. In this context, several studies have shown that recognition of the molecules derived from *Leishmania* is achieved by different TLRs, including TLR2, TLR3 and TLR4 [29–32]. The main PAMPs present on the *Leishmania* surface and recognized by these receptors on cells of the immune system include lipophosphoglycan (LPG), glycosylinositolphospholipids (GIPL) and gp63 [29,30,33]. Accordingly, it was demonstrated that LPG from *Leishmania major* stimulated macrophages and NK cells to secrete cytokines such as IL-12, TNF-α and IFN-γ by binding to TLR2 [29,30]. Notably, TLR4-deficient mice are unable to control *L. major* replication and consequently develop lesions that are more severe compared to control animals [34]. Accordingly, *in vitro* studies conducted by Kropf *et al*. [31] demonstrated a role for TLR4 in the control of *L. major* infection related to increased expression of inducible nitric oxide synthase (iNOS). Studies conducted in BALB/c mice infected with *L. chagasi* demonstrated increased TLR2 and TLR4 mRNA expression in the spleen, which was correlated with parasite load [35]. Murine macrophages stimulated with IFN-γ were also shown to recognize and phagocytose *L. donovani* with consequent production of TNF-α and NO via TLR2 and TLR3 signaling [32]. Das *et al*. [36] reported that overexpression of TGF-β1 downregulates the expression of TLR4 on *L. donovani*-infected macrophages obtained from patients with VL.

Despite all studies cited above, there is still a lack of information about the involvement of pro- and anti-inflammatory cytokines in susceptibility to or protection against VL, as well as the involvement of different TLRs in their production. Furthermore, it should be noted that studies evaluating the production of these cytokines by cells of pre- and post-treatment patients are scarce, and investigation of these aspects could contribute to a better understanding of the parasite/host relationship in patients with VL. Therefore, the current study evaluated the surface expression of TLR2 and TLR4 in monocytes and lymphocytes as well as the involvement of these receptors in the production of TNF-α, IFN-γ, IL-17, TGF-β, IL-10 and NO in patients with VL before and after treatment.

## Materials and Methods

### Patients

A total of 13 newly diagnosed VL patients were evaluated before and after treatment. These patients had never presented the disease before nor did they relapse after treatment. The patients consisted of both males and females, although the majority were male (11 individuals), with ages ranging from 18 to 53 years (see Table 1). Patients diagnosed with other infectious or granulomatous diseases, as well as HIV-infected patients and pregnant women, were excluded. Patients were enrolled in the study at the time they contacted the health care service for diagnosis and treatment of the disease. Patients were recruited from Bauru State Hospital (*Hospital Estadual de Bauru*) and the Clinic Hospital of Marilia (*Hospital das Clínicas de Marília*) in the state of Sao Paulo, Brazil. A total of 16 gender- and age-matched healthy individuals were evaluated as controls.

All patients and control individuals who agreed to participate in the study were informed about the study and signed a free and informed consent form. This study was approved by the Research Ethics Committees of the Botucatu Medical School (*Faculdade de Medicina de Botucatu*), Bauru State Hospital and Clinic Hospital of Marilia and was performed in accordance with the principles of the Declaration of Helsinki, 1964.

**Table 1:** 

	Patients *(n = 13)*	Control *(n = 16)*
Age—Mean (±SD)	36,3 (±11,6)	35,9 (±11,0)
Gender—M/F	11/2	13/3
Completed High School—n (%)	3 (23,1%)	16 (100,0%)
Smoking habit—n (%)	10 (76,9%)	0 (0%)
Treatment with Glucantime* - n (%)	2 (15,4%)	-
Treatment with Ampho B** - n (%)	4 (30,8%)	-
Treatment with Ampho L*** - n (%)	7 (53,8%)	-

* *Glucantime (M-metyl-glucamine)scheme*: *20 mg/Kg/day for 20 days*

** *Deoxycholate Amphotericin B scheme*: *1 mg/Kg/day for 20 days*

*** *Liposomal Amphotericin B scheme*: *5 mg/Kg/day for 5 days*

### Diagnosis and treatment of VL

The diagnosis of VL was performed through the confirmation of parasites in bone marrow aspirates. Patients were treated with N-Methylglucamine antimoaniate, amphotericin B or liposomal amphotericin B according to the judgment of the physician assistant (Table 1). Patient samples were collected at the hospital before the initiation of VL treatment and 1 to 3 months after completion of the drug regimen when the patients returned to the hospital for follow-up care. According to the laboratory and clinical tests, all patients were successfully treated.

### Blood sample collection

Blood samples (25 ml) were taken from a forearm vein once in control individuals and twice in patients (before and after treatment). Blood was collected in heparinized tubes and then centrifuged at 450 g for 10 minutes. Blood samples were used to evaluate cell-surface expression of TLR2 and TLR4 via flow cytometry, to obtain peripheral blood mononuclear cells (PBMCs) and for measure cytokines in culture supernatants.

### Cell-surface expression of TLR2 and TLR4

To evaluate the expression of TLR2 and TLR4 on monocytes and lymphocytes, 100 μl of whole blood from patients and control individuals was incubated with an anti-human CD3 monoclonal antibody conjugated with PE-DY647 (EXBIO, Vestec, Czech Republic), an anti-human CD14 monoclonal antibody conjugated with PE-DY647 (EXBIO, Vestec, Czech Republic), an anti-human TLR4 antibody conjugated with FITC (Biolegend, San Diego, CA, USA) and an anti-human TLR2 antibody conjugated with PE (Biolegend, San Diego, CA, USA) in falcon tubes (Becton, Dickinson and Company, Franklin Lakes, NJ, USA) for 20 minutes in the dark. After incubation, the cells were washed with 450 μl of red blood cell (RBC) lysis solution (Becton, Dickinson and Company, Franklin Lakes, NJ, USA) and incubated for 15 minutes in the dark. Then, the samples were centrifuged for 5 minutes at 450 g, and the supernatant was discarded. This procedure was performed twice. Cells were suspended in 300 μl of ISOTON II electrolyte solution (Becton, Dickinson and Company, Franklin Lakes, NJ, USA). For each test, there was a control tube in which cells were incubated with isotype control antibodies conjugated with the same fluorochromes used in the test. Analysis and cell acquisition were performed via flow cytometry (FACSCalibur^TM^, Becton, Dickinson and Company, Franklin Lakes, NJ, USA) using the Cell Quest software (Becton, Dickinson and Company, Franklin Lakes, NJ, USA). Concerning the gating strategies applied to distinguish between cells populations analyzed via flow cytometry, FSC-SSC profile was used to distinguish total lymphocytes and monocytes and this subtype of cells were gated according to light scatter profile and the expression of CD3 and CD14. Acquisition was standardized to 10.000 events per sample.

### PBMCs isolation

PBMCs were obtained using the Histopaque gradient method (Sigma-Aldrich, St. Louis, MO, USA). The ring rich in lymphocytes and monocytes was removed and washed with RPMI-1640 (Sigma-Aldrich, St. Louis, MO, USA) and then centrifuged at 450 g for 15 minutes. The cells were subsequently resuspended in complete medium, consisting of RPMI-1640 supplemented with 2 mM L-glutamine, 40 μg/ml gentamicin and 10% fetal bovine serum (Nutricell, Campinas, São Paulo, Brazil). Identification of the cells and assessment of their viability were performed using Trypan Blue staining and cell concentration was adjusted to 1 x 10^6^ cells/ml to perform the other protocols.

### Stimulation of PBMCs

PBMCs (1 x 10^6^ cells/ml) in complete medium were plated in 24-well culture plates in the absence or presence of TLR agonists, according to the manufacturer’s recommendations (Invivogen; San Diego, CA, USA). The following concentrations of agonists were used: 5 μg/ml peptidoglycan (PGN) from *Staphylococcus aureus* (TLR2 agonist) and 1 μg/ml ultra-pure lipopolysaccharide (LPS) from *Escherichia coli* K12 (TLR4 agonist). PBMCs were incubated with the agonists at 37°C under 5% CO_2_ for 24 hours. After incubation, the supernatants were aspirated, aliquoted and stored at −80°C prior to analysis.

### Cytokine production

The cytokines TNF-α, IFN-γ, IL-17, IL-10 and TGF-β were measured in culture supernatants using the CBA (Cytometric Beads Array) technique and analyzed via flow cytometry using a FACSCalibur (Becton, Dickinson and Company), Cell Quest Software and FCAP Array Software (Becton, Dickinson and Company, Franklin Lakes, NJ, USA) according to the manufacturer’s instructions. The limit of detection of cytokines for TNF-α, IFN-γ, IL-17, IL-10 and TGF-β were 0,7 pg/ml, 1,8 pg/ml, 0,3 pg/ml, 0,13 pg/ml and 14,9 pg/ml respectively. The limit of detection was determined by evaluating the estimated average mean fluorescence intensity (MFI) in negative control (0 pg/ml) + 2 standard deviation, and results below the limits of detection were computed as 0.

### Detection of NO

The NO levels were analyzed in supernatants from PBMCs cultures, measuring the levels of nitrite/nitrate using a commercial colorimetric assay kit (Cayman Chemical Company, Ann Arbor, MI, USA) according to the manufacturer’s instructions. The kit provides an accurate and convenient method for the measurement of NO, first based on the conversion of nitrate utilizing nitrate reductase, and after addition the Griess reagents, which convert nitrite into a deep purple azo compound. The present data are representative of three replicates.

### Statistical analysis

Fisher’s exact test was used to analyze non-continuous variables. Negative binomial regression was applied for data that exhibited extra variation, as this type of distribution can estimate variance that cannot be identified through the Poisson regression. After applying negative binomial regression, dependent variables were calculated using repeated measures tests, and independent variables were calculated through comparison of means. The Friedman test was employed to analyze asymmetric data between dependent groups. Asymmetric data between two dependent groups were analyzed with the Wilcoxon test, and such data between two independent groups with analyzed with the Mann-Whitney test. All analyses were performed using the Statistical Analysis System (SAS) software v.9.3. The significance level was set at 5% or the corresponding p-value.

## Results

### Expression of TLR2 and TLR4 on monocytes and lymphocytes

**Fig 1:**
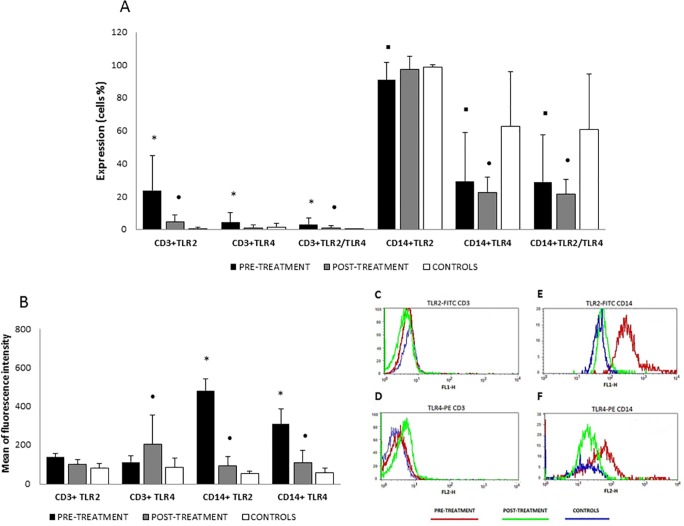
Expression of surface TLR2 and TLR4. Frequency of CD3^+^ and CD14^+^ cells expressing TLR2, TLR4 and co-expressing TLR2 and TLR4 (A) and mean of fluorescence intensity (B) of TLR2 and TLR4 in CD3^+^ and CD14^+^ cells in whole blood from patients with visceral leishmaniasis pre-treatment, post-treatment and control subjects. Gating strategies to distinguish between cell populations analyzed by flow cytometry. FSC-SSC profile was used to distinguish total lymphocytes and monocytes and these subtype cells were gated according to light scatter profile and the expression of CD3 or CD14. Representative histograms plots of TLR2 and TLR4 expression in CD3^+^ (C,D) and CD14^+^ (E,F) cells. The results are expressed as the mean and standard deviation. *p<0.05 compared pre-treatment *vs*. post-treatment and control subjects; ▪ p<0.05 compared pre-treatment *vs*. control subjects and • p<0.05 compared post-treatment *vs*. control subjects.

The expression and coexpression of TLR2 and TLR4 on the surface of lymphocytes and monocytes was assessed by measuring the percentage of CD3^+^ and CD14^+^ cells positive for TLR2 and TLR4 in patient samples before and after treatment. After selection of the populations of interest (CD3^+^ and CD14^+^), TLR2 and TLR4 expression and the MFI were evaluated, with the threshold being based on the respective isotype controls (Fig. 1). The results concerning TLR2 and TLR4 surface expression demonstrated that patients presented higher percentages of CD3^+^ cells expressing TLR2 and TLR4, as well as cells coexpressing TLR2/TLR4 before treatment compared with post-treatment and control individuals (p<0.05). After treatment, percentage of CD3^+^ cells expressing these receptors was significantly reduced, though patients still showed higher expression of TLR2 and coexpression of TLR2/TLR4 compared with control individuals (p<0.05). There was no significant difference in the expression of TLR4 between post-treatment patients and control individuals (Fig. 1A). Regarding the MFI (Fig. 1B and C), there were no significant differences in the expression of TLR2 or TLR4 in CD3^+^ cells from pre-treatment patients compared with post-treatment patients and control individuals. However, the fluorescence intensity of TLR4 expression in CD3^+^ cells was higher after treatment when compared to the controls (p<0.05).

In relation of monocytes (CD14^+^), there were no significant differences in percentage of cells expressing TLR2, TLR4 and coexpressing TLR2/TLR4 between patients pre- and post-treatment. However, patients with active VL presented a lower percentage of cells expressing TLR2, TLR4 and coexpressing TLR2/TLR4 (p<0.05) compared to control individuals (Fig. 1A). Furthermore, the MFI of TLR2 and TLR4 in pre-treatment patients was significantly higher (p<0.05) compared with post-treatment patients and control individuals (Fig. 1B, C). After treatment, although the patients still presented lower percentages of CD14^+^ cells expressing TLR4 and co-expressing TLR2/TLR4 (p<0.05) compared with control individuals (Fig. 1A), the fluorescence of TLR2 and TLR4 on monocytes remained higher (p<0.05) compared with control individuals (Fig. 1B, C).

### Involvement of TLR2 and TLR4 in cytokine production

**Fig 2:**
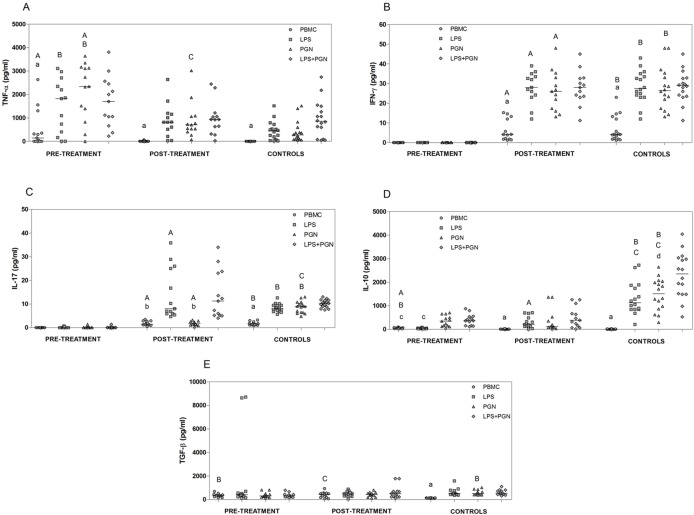
Cytokine production after stimulation with LPS and PGN agonists. Levels of TNF-α (A), IFN-γ (B), IL-17 (C), IL-10 (D) and TGF-β (E) in supernatants were analyzed after 24 hours of PBMCs cultured (1x10^6^ cells/ml), obtained from patients with VL pre-treatment, post-treatment and control subjects, and stimulated or not with LPS ultra-purified (1 μg/ml), PGN (5 μg/ml) and LPS+PGN. Lowercase letters represent significant differences among agonists in the same group: p<0.05 **a**—PBMC *vs*. LPS, PGN, LPS+PGN; **b**—PBMC, PGN *vs*. LPS, LPS+PGN; **c**—PBMC, LPS *vs*. PGN, LPS+PGN; **d**—LPS *vs*. LPS+PGN. Capital letters represent significant differences in the same agonists among groups: p<0.05 **A**—pre-treatment *vs*. post-treatment; **B**—pre-treatment *vs*. control individuals; **C**—post-treatment *vs*. control individuals. Cytokine levels were measured by CBA. Each dot represent a different patient and each bar represents the median.

In the next set of experiments, we evaluated the involvement of TLR2 and TLR4 in the production of pro- and anti-inflammatory cytokines by PBMC from patients with VL pre- and post-treatment (Fig. 2). For this, the PBMC cultures from pre- and post-treatment patients and control individuals were either stimulated with PGN (TLR2 agonist) and LPS (TLR4 agonist), or left unstimulated. Then, cytokines levels were evaluated in the culture supernatants and analyzed both between different groups (each stimulus *versus* groups) and separately by group (each group *versus* stimuli). Unstimulated PBMCs from pre-treatment patients produced significantly higher levels of TNF-α (p = 0.02) and IL-10 (p<0.001) and lower levels of IFN-γ (p<0.001) and IL-17 (p<0.001) compared with samples obtained post-treatment (Fig. 2A-D). There was no difference in TGF-β levels in unstimulated PBMCs from patient samples obtained pre- and post-treatment (Fig. 2E). Following stimulation with PGN, cells from pre-treatment patients produced significantly higher levels of TNF-α (p = 0.01) in relation to post-treatment patients, and upon stimulation with LPS, PBMCs from pre-treatment patients produced lower levels of IL-10 (p<0.001) in relation to post-treatment patients. Furthermore, PBMCs from pre-treatment patients stimulated with LPS or PGN released significantly lower IFN-γ and IL-17 levels (p<0.001) compared with those obtained from post-treatment patients (Fig. 2A-D).

Unstimulated cells from pre-treatment patients released significantly lower levels of IFN-γ and IL-17 (p<0.001) and significantly more IL-10 (p<0.001) compared with those obtained from control individuals (Fig. 2B, C, D). Moreover, unstimulated PBMCs from pre- and post-treatment patients produced significantly higher levels of TGF-β (p<0.001, p = 0.01 respectively) compared with cells from control individuals (Fig. 2E). Interestingly, when we compared cytokine production by PBMCs stimulated with LPS or PGN between patients and control individuals, it was evident that cells from pre-treatment patients produced significantly higher levels of TNF-α (p<0.001) and lower levels of IFN-γ and IL-17 (p<0.001) compared with those obtained from control individuals (Fig. 2A, B, C). PBMCs from pre- and post-treatment patients produced significantly lower levels of IL-10 (p<0.001) compared to those obtained from control individuals (Fig. 2D). PBMCs from pre-treatment patients stimulated with PGN produced significantly lower TGF-β levels (p = 0.008) than those of control subjects (Fig. 2E). PBMCs from post-treatment patients stimulated with PGN released higher levels of TNF-α (p = 0.02) and lower levels of IL-17 (p<0.001) compared with those from control individuals (Fig. 2A, C).

Through analyzing the involvement of TLRs in the production of cytokines in the studied groups, we found that cells stimulated with LPS, PGN or LPS+PGN in all groups produced significantly higher levels of TNF-α (p<0.05) compared with unstimulated cells (Fig. 2A). Furthermore, in patients with active VL, no significant differences in IFN-γ and IL-17 production were found between PBMCs stimulated with agonists or left unstimulated. However, stimulation with all of the tested agonists significantly increased the production of IFN-γ (p<0.05) by PBMCs from post-treatment patients and control individuals and the production of IL-17 (p<0.05) in cells from control individuals compared with unstimulated cells (Fig. 2B, C). Moreover, cells from post-treatment patients stimulated with LPS or LPS+PGN produced significantly higher levels of IL-17 (p<0.05) compared with unstimulated cells and stimulated with PGN (Fig. 2C). Regarding IL-10, PBMCs from pre-treatment patients stimulated with PGN or LPS+PGN presented higher levels of IL-10 (p<0.05) compared with unstimulated cells and cells stimulated with LPS. In post-treatment patients and in control individuals, there was a significant increase in IL-10 (p<0.05) when cells were stimulated with all agonists compared with unstimulated cells. In addition, in control individuals, stimulation with LPS+PGN resulted in higher levels of IL-10 (p<0.05) compared with stimulation with LPS (Fig. 2D). PBMCs from pre- and post-treatment patients showed no significant difference in the production of TGF-β, regardless of whether they were stimulated with TLR agonists, whereas in control individuals, stimulation with all agonists increased the production of TGF-β (p<0.05) compared with unstimulated cells (Fig. 2E).

### Involvement of TLR2 and TLR4 in the production of NO

**Fig 3:**
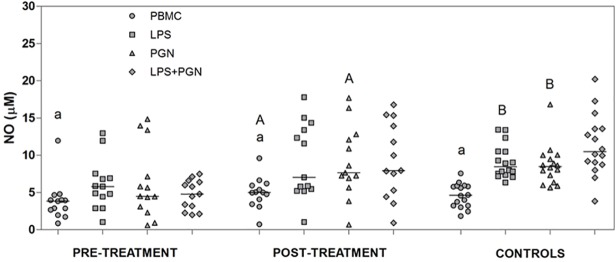
NO production after stimulation with LPS and PGN agonists. Levels of NO in supernatants were analyzed after 24 hours of PBMCs cultured (1x10^6^ cells/ml), obtained from patients with VL pre-treatment, post-treatment and control subjects, and stimulated or not with LPS ultra-purified (1 μg/ml), PGN (5 μg/ml) and LPS+PGN. Lowercase letters represent significant differences among agonists in the same group: p<0.05 **a**—PBMC *vs*. LPS, PGN, LPS+PGN. Capital letters represent significant differences in the same agonists among groups: p<0.05 **A**—pre-treatment *vs*. post-treatment; **B**—pre-treatment *vs*. control individuals. Each dot represent a different patient and each bar represents the median. NO levels were measured by the levels of nitrite/nitrate and the data are representative of triplicates.

NO production was measured in the supernatants of cultures of PBMC from patients with VL pre- and post-treatment (Fig. 3). We demonstrated that unstimulated cells obtained from patients prior to treatment produced significantly lower levels of NO (p = 0.04) compared with cells obtained post-treatment. When LPS and PGN were used as stimuli, PBMCs from pre-treatment patients produced lower NO levels (p = 0.002, p = 0.02) compared with control individuals. Furthermore, PBMCs from pre-treatment patients stimulated with PGN produced lower NO levels (p = 0.03) than PBMCs from post-treatment patients. The involvement of TLR2 and TLR4 in NO production following stimulation with the agonists PGN and LPS, respectively, was also verified. The results showed that stimulation with LPS, PGN and LPS+PGN significantly increased (p<0.05) the levels of NO compared with unstimulated cells in pre- and post-treatment patients and control individuals, suggesting that both TLR2 and TLR4 are involved in the production this metabolite.

## Discussion

To our knowledge, this is the first study to assess the involvement of TLR2 and TLR4 in NO and pro- and anti-inflammatory cytokine production by VL patients before and after treatment with anti-leishmanial drugs. In this work, the ability of PBMCs to produce these cytokines and NO were evaluated upon stimulation with TLR2 and TLR4 agonists. In addition, we determined whether the obtained results were associated with the capacity of lymphocytes and monocytes of these two groups to differentially express TLR2 and TLR4. It is noteworthy that the production of cytokines and NO by PBMCs was evaluated without distinction between monocytes and lymphocytes, and it is known that these cells differ in their ability to produce these mediators. Thus, differences in the proportion of monocytes and lymphocytes between the groups can affect the amount of cytokines and NO produced in the culture supernatant. Hence, we did not find significant differences in the proportion of CD3^+^/CD14^+^ among the groups (data not shown).

Both lymphocytes and monocytes from patients pre- and post-treatment expressed TLR2 and TLR4, and these results are in agreement with those of other studies [31–35], showing the involvement of these receptors in the recognition of *Leishmania* [37]. Pre-treatment patients exhibited an increased number of lymphocytes expressing TLR2 and TLR4 and after treatment, they expressing TLR2 and TLR2/TLR4. Moreover, after treatment, even with the reduced number of lymphocytes expressing TLR4, the MFI of this receptor was higher, indicating that although the number of cells expressing this receptor was reduced, the expression of TLR4 was increased in these cells. TLRs play an important role in the immune response, as their ligands can modulate the immune functions of lymphocytes. For example, TLRs expressed on lymphocytes can act as co-stimulatory receptors of the T cell receptor (TCR) to enhance proliferation and/or cytokine production [38–41]. On monocytes, TLR ligands can have a variety of effects, such as the induction and production of cytokines and expression of major histocompatibility complex (MHC) II molecules [42]. Experimental studies on *L. donovani* showed that macrophages are extremely important in the immune response because they express high levels of TLR2 and TLR4, which leads to TNF-α and NO production and parasite clearence [32,43]. Regarding the expression of TLR2 and TLR4 in monocytes, although pre- and post-treatment patients presented lower percentage of cells expressing these receptors, the MFI of TLRs was higher. Taken together, our results demonstrated the expression of TLR2 and TLR4 during active VL as well as after treatment, suggesting that these receptors may act in both pathogenic and protective mechanisms through the induction of pro- and anti-inflammatory mediators.

The results of the current study demonstrated that patients with active VL produced higher levels of TNF-α, which were decreased after treatment, these data are in agreement with some previous studies [44–46] but differ from others [47–49]. The production of this cytokine in the evaluated groups was associated with the expression of TLR2 and TLR4. Similarly, these receptors were to be involved in TNF-α production in studies on hepatitis C [50] and *L. donovani* [32,51]. In contrast, Murray *et al*. [37] observed that only TLR4, and not TLR2, was related to the production of TNF-α in the liver of C57BL/6 mice infected with *L. donovani*. Furthermore, the association of TLR2 and TLR4 with TNF-α production was most apparent in patients with active VL as the agonists stimuli increased production compared with the other groups. After treatment, the reduced expression of TLRs could at least partially explain the reduction of TNF-α levels in this group, and these low levels could be associated with protection, i.e., parasite clearance mediated by TLR2 and TLR4 [32,51]. An recent study revealed that the anti-leishmanial drugs, miltefosine and paramomycin, induce release of TNF-α in a TLR4-dependent manner [52]. TNF-α is a cytokine involved in the control of infection and is an important factor associated with the immunopathogenesis of disease [53–56]. However, in the current study, despite the higher levels of TNF-α found in patients with active VL we were not able to elucidate whether this cytokine was protective or was involved in disease pathogenesis.

IFN-γ plays an essential role in macrophage-mediated anti-leishmanial activity, contributing to parasite elimination and the subsequent resolution of infection [12,14,20,57]. Data from the current study showed that cells from patients with active VL did not produce IFN-γ, unlike the post-treatment patients and control individuals. Similarly, children from an endemic area of VL who presented low levels of IFN-γ showed disease progression and this low levels of IFN-γ were associated with a high parasite load [15,16]. On the other hand, children infected with *L. chagasi* were found to be able to control the infection by generating high levels of IFN-γ [16]. In contrast to our results, Duthie *et al*. [27] reported that patients with active VL exhibited high levels of IFN-γ, which were decreased after treatment. We did not observe involvement of TLR2 and TLR4 in the production of IFN-γ in patients with active VL, however, an experimental study on *L. donovani* showed that IFN-γ production by TLR4 is essential for protection [37]. Furthermore, IFN-γ levels in patients with active VL were very low despite the fact that they express higher levels of TLRs, suggesting that other factors are influencing the non-production of this cytokine. An unknown mechanism may occur in these patients that likely prevents the binding of agonists to the receptors expressed on the cell surface. On the other hand, we observed an association of TLR2 and TLR4 in IFN-γ production in patients after treatment. Increased expression of TLR4 in PBMCs and subsequent production of IFN-γ and iNOS were observed in a study in which VL patients treated with miltefosine [51]. Our results suggest that the increased levels of IFN-γ after treatment may have driven the protective response, and this mechanism involved the activation of TLR2 and TLR4.

Th17 cells are involved in the development of inflammatory and autoimmune diseases as well as protection against some intracellular pathogens [58–60], including *Leishmania* [28]. In cutaneous leishmaniasis, high levels of this cytokine are associated with tissue destruction, inflammation and disease pathogenesis [25,26]. However, the precise role of Th17 cells in VL, especially in patients, is not entirely clear. The presented work is a pioneering study relating the expression of TLRs and IL-17 production in patients with VL. The pre-treatment patients showed a similar profile to that detected for IFN-γ production, and cells from patients with active VL did not produce IL-17, even after stimulation. Similar results were obtained in patients with VL in another study [27]. On the other hand, infection with *L. donovani* led to a greater Th17 cells differentiation and higher levels of IL-17, which was associated with protection [28]. Patients with indeterminate or mild cardiac forms of Chagas disease, also displayed high levels of IL-17 in either the absence or presence of mild cardiac lesions, demonstrated the protective role of this cytokine in another type of parasitic infection [61]. Our results showed increased levels of this cytokine after treatment, in accord with an experimental study in which treatment with curdlan was also able to induce the production of cytokines such as IL-17 and IL-23, thereby contributing to the reduction of parasite load [62]. However, it was not possible to determine whether the increase of IL-17 after treatment contributed to reduce the parasitic load and controlling infection. Additionally, our results demonstrated that low levels of IL-17 in pre-treatment patients were not associated with TLR2 or TLR4, although an association between TLR4 and IL-17 was observed after treatment, considering that stimulation with LPS increased the production of this cytokine. In agreement with the current study, an experimental model using *L. chagasi* also showed an association between IL-17 and TLR4 [35]. Furthermore, an interaction between TLR4 and IL-17 has been demonstrated in other infections and comorbidities [63,64]. Thus, our results suggest that TLR4 is involved in IL-17 production.

NO is produced during active leishmaniasis and plays an important role in controlling the parasite burden and contributing to disease prevention [65–68]. However, according to the current results, NO production in patients prior to treatment was low and increased following treatment. Similarly, Kumar *et al*. [69] found that monocytes from patients with VL produced low levels of NO before treatment, whereas the levels of this metabolite were increased at the end of treatment. After treatment, we observed an increase in IL-17, IFN-γ and NO levels. Accordingly, a recent study demonstrated that IL-17 acts synergistically with IFN-γ to potentiate NO production and leishmanicidal activity in infected macrophages [70]. Analysis of the involvement of TLR2 and TLR4 in NO production demonstrated that these two receptors appeared to be involved in NO production in all groups. *In vitro* studies with *Leishmania* further showed that NO production was associated with the same receptors [32,71]. Therefore, we can suggest that before treatment, the parasite activates suppressor mechanisms via TLR2 and TLR4 that can decrease the production of NO. After treatment, we noted increased levels of NO and possible involvement of TLR2, mainly in relation to its production. A previous study showed that neutralization of TLR4, but not of TLR2, damaged the production of NO from *L. donovani*-infected macrophages treated with anti-leishmanial drugs [52]. Moreover, increased levels of factors that activate macrophages, such as IFN-γ, may also have contributed to the NO increased levels.

Similar to other infections, IL-10 and TGF-β have been identified as important cytokines in leishmaniasis that are involved in homeostatic mechanisms and the control of tissue damage caused by excessive inflammation [72–76]. On the other hand, these cytokines also favor the persistence of the pathogen by disabling the functions of macrophages and suppressing the production of IL-12, IFN-γ, TNF-α and NO [11,13,77–81]. The current results showed that patients with active VL produced high levels of IL-10, which is in agreement with other studies [82,83]. Furthermore, the levels of IL-10 detected pre-treatment were associated with the expression of TLR2. Similarly, a study conducted by Chandra *et al*. [84] demonstrated that human monocytes infected with *L. donovani* were able to modulate the immune response through TLR-2 and IL-10. Another study demonstrated that pre-treatment with an anti-TLR2 antibody decreased IL-10 expression in *L. donovani*-infected human macrophages, showing that TLR2 may be related to regulation of the immune response [55]. After treatment, the production of IL-10 was associated with TLRs, and mainly with TLR4. Likewise, macrophages infected with *L. amazonensis* and *L. major* exhibited greater production of this cytokine when they were stimulated with LPS [85]. In the current study, untreated patients presented reduced levels of IFN-γ and IL-17, and high levels of IL-10 associated with TLR2, whereas after treatment, there was a reduction in IL-10 production and increased levels of IFN, IL-17 and NO. This result suggests that the production of IL-10 through TLR2 before treatment may be responsible for inhibiting the protective mechanisms of the immune response.

The high levels of TGF-β detected pre-and post-treatment were associated with low levels of IFN-γ, IL-17 and NO before treatment and with high levels of these mediators after treatment. Considering these results, we suggest that TGF-β production is associated with immunosuppressive mechanisms during active disease, whereas after treatment, this cytokine may contribute to the increase in IL-17, as it is well established that TGF-β along with IL-6 is involved in the induction of Th17 cells [86,87]. Some studies have also shown that BALB/c mice infected with *L. chagasi* display impaired Th1 immune responses due to the increased production of TGF-β by CD4^+^ T cells [88]. Furthermore, the current study showed that the production of TGF-β did not involve TLR2 and TLR4, suggesting that other receptors may have mediated its production. Similar our results, Das *et al*. [36], showed that macrophages from patients with VL do not produce TGF-β1 when stimulated with TLR2 and TLR4 agonists, however, increased levels of TGF-β1 decreases TLR4 expression.

There are some limitations of our study that deserve comments. First, given the small sample size, our results need to be confirmed in the future through larger cohort studies. Second, the timing of blood sample collection from patients after treatment ranged from 1 to 3 months due either to the therapeutic scheme or to the date of return of patients to the hospital for follow-up care. The majority of patients did not live in the same city as the hospital, and the date of return was dependent not on the researchers but on the judgment of the hospital and the doctors. These factors could theoretically bias our results. Although we evaluated the involvement of TLR2 and TLR4 in the production of cytokines and NO in VL patients before and after treatment using agonists of these receptors, further studies are necessary to corroborate these results using *Leishmania*-derived TLR agonists, as it is known that not all agonists acting through the same TLR share the same effects.

## Conclusions

According to the data obtained the present study, pre-treatment patients showed expression of TLRs, increased levels of TNF-α, IL-10 and TGF-β and decreased levels of IFN-γ, IL-17 e NO. Furthermore, the involvement of TLR2 and TLR4 in the production of TNF-α and NO and involvement of TLR2 in the production of IL-10 were observed. After treatment, when the infection had been controlled, the expression of TLR2 and TLR4 was still detected, and these TLRs may have been involved in the production of TNF-α, IFN-γ, IL-10 and NO, while TLR4 was involved in the production of IL-17. The TGF-β production was not associated with any TLRs. These results suggest that both receptors were involved in cytokines and NO production before and after treatment, leading to the immunopathogenesis observed during active VL and the induction of protective mechanisms after treatment.
